# Multiple Brain Abscesses Due to Nocardia otitidiscaviarum: Case Report and Treatment Implications

**DOI:** 10.7759/cureus.14362

**Published:** 2021-04-08

**Authors:** Evan H Einstein, David Bonda, Salman Khan, Avraham B Zlochower, Randy S D'Amico

**Affiliations:** 1 Neurosurgery, Lenox Hill Hospital/Donald and Barbara Zucker School of Medicine at Hofstra, New York, USA; 2 Infectious Disease, Lenox Hill Hospital/Donald and Barbara Zucker School of Medicine at Hofstra, New York, USA; 3 Radiology, Lenox Hill Hospital/Donald and Barbara Zucker School of Medicine at Hofstra, New York, USA

**Keywords:** nocardia, brain abscess, nocardia otitidiscaviarum, intrathecal antibiotics, decompressive hemicraniectomy

## Abstract

*Nocardia* infections typically present in immunocompromised hosts. Brain abscesses caused by species such as *Nocardia asteroides, farcinica, *and* abscessus* are well-documented in the literature. We present a rare case of an immunocompetent patient with multiple brain abscesses due to *Nocardia otitidiscaviarum* requiring a decompressive fronto-temporoparietal craniectomy due to symptomatic intracranial hypertension. The patient was treated with intrathecal amikacin in addition to standard antibiotics with the resolution of the disease and good neurologic outcome. This is one of few case reports overall involving this species within the brain, and the second to report favorable outcomes. This case describes implications for treatment and adds to sparse literature regarding this particular pathogen.

## Introduction

*Nocardia* is a genus representing a ubiquitous group of aerobic, gram-positive bacteria typically causing opportunistic infections in immunocompromised hosts [1]. These infections typically occur in the pulmonary system, with the central nervous system (CNS) being the most common site of extrapulmonary nocardiosis [2]. Although nocardial brain abscesses often occur in immunocompromised patients [3-4], multiple case reports have demonstrated these abscesses in immunocompetent patients as well [5-10]. *Nocardia otitidiscaviarum* is a relatively rare species occurring in approximately 3% of overall general *Nocardia* cases [11], with only three published reports describing brain abscesses in immunocompetent hosts [12-14]. We present a case of an immunocompetent patient with multiple brain abscesses caused by *Nocardia otitidiscaviarum*. The patient was treated with decompressive craniectomy due to symptomatic intracranial hypertension and a combination of systemic and intrathecal antibiotic therapy with resolution of the disease and good neurologic outcome. This is the second report of a favorable outcome in an immunocompetent individual and describes an aggressive treatment strategy that may have implications on future therapy for this rare disease.

## Case presentation

A 46-year-old man with a history of controlled type 2 diabetes (A1C 6.8 percent on admission), employed as a landscaper, presented to an outside hospital with intermittent fevers, chills, weight loss, and generalized weakness. He was found to have bilateral pneumonia and underwent a bronchoscopy yielding biopsies with negative cultures, negative fungal and acid-fast bacilli (AFB) stains, and pathology consistent with inflammation. He was treated with a course of amoxicillin-clavulanate and discharged with a plan to follow up with outpatient pulmonology given the ongoing uncertainty of his pulmonary diagnosis.

He presented to our institution two weeks later after a new-onset generalized tonic-clonic seizure. The patient had been experiencing a frontal headache with increasing aggression and confusion the day prior to presentation. Upon arrival, the patient was afebrile and hemodynamically stable. Laboratory values demonstrated a white blood cell (WBC) count of 8.45 x 10^9 ^cells/L, red blood cell (RBC) count of 3.78 x 10^12 ^cells/L, platelets of 144 x 10^9 ^cells/L, absolute cluster of differentiation 3 (CD3) count of 612 cells/µL, and absolute CD4 count of 239 cells/µL. He was human immunodeficiency virus (HIV) negative. His neurologic exam demonstrated an altered sensorium, as he was only oriented to self and intermittently attentive to the interview. He otherwise did not demonstrate any focal neurologic deficits and all cranial nerves appeared to be intact.

**Figure 1:**
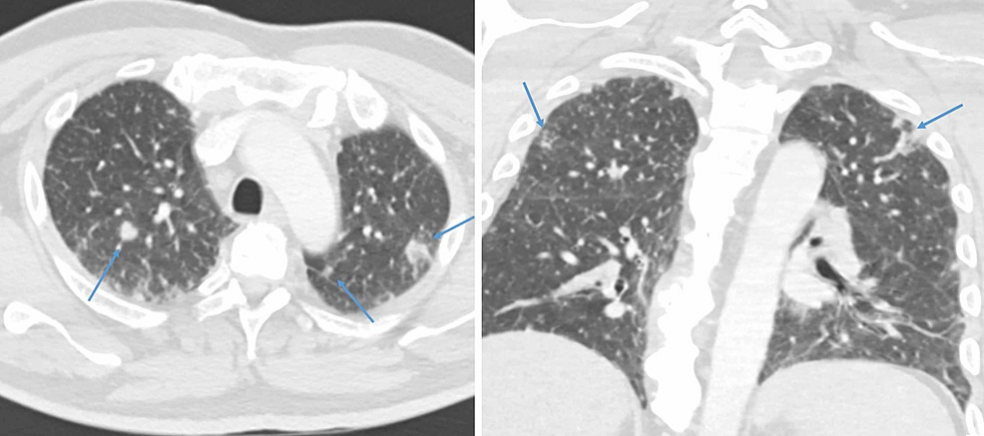
Axial and coronal CT chest images demonstrating numerous pulmonary nodules

**Figure 2:**
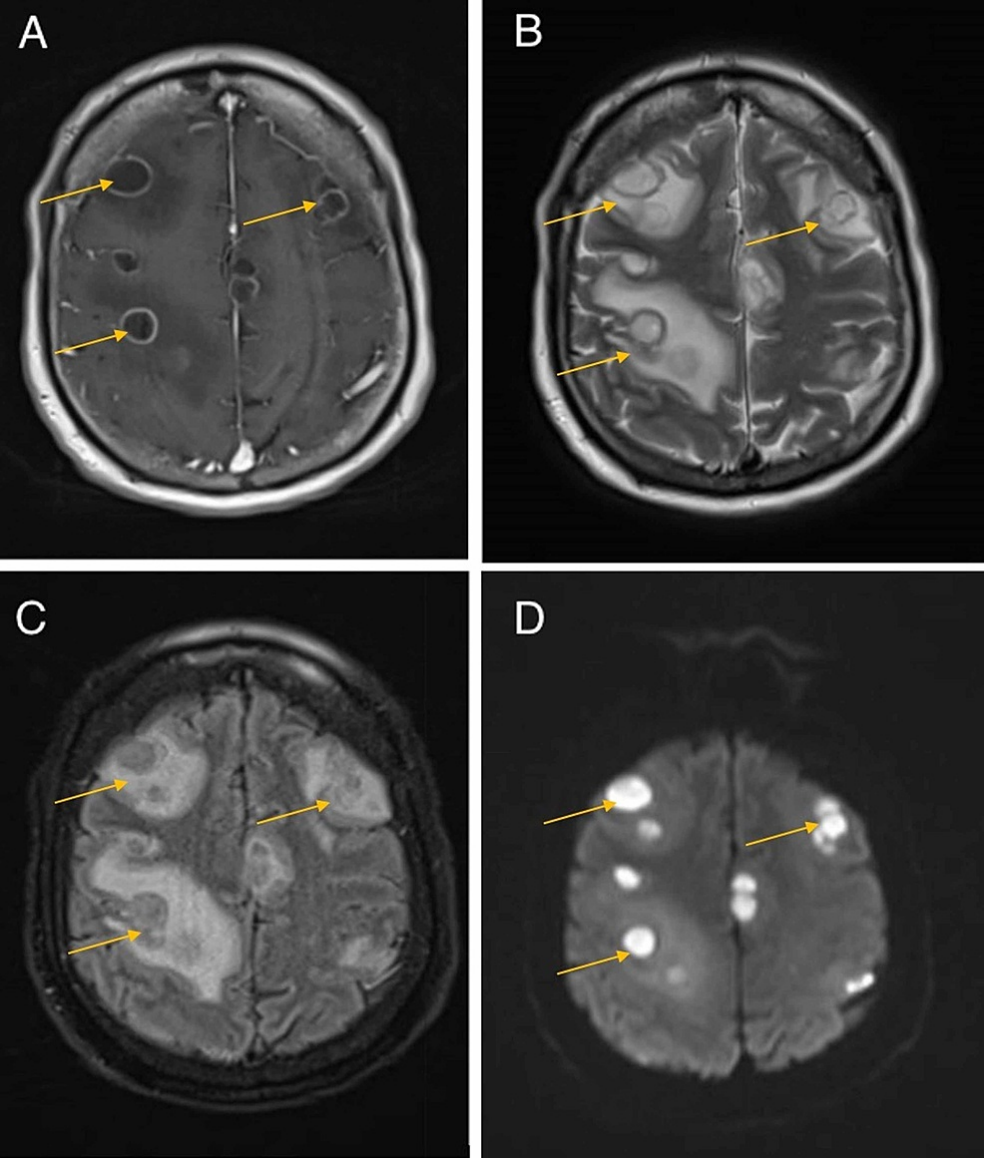
Initial MR brain imaging of the patient following new-onset tonic-clonic seizure (A) T1-weighted post-contrast imaging demonstrating multiple ring-enhancing lesions. (B) T2-weighted image demonstrating multiple hyperintense lesions with surrounding hyperintensity due to vasogenic edema. (C) T2-weighted-fluid-attenuated inversion recovery (T2-FLAIR) image demonstrating isointense signal within the lesions with surrounding hyperintensity due to vasogenic edema. (D) Diffusion-weighted imaging (DWI) restricted diffusion within the lesions consistent with an abscess.

The patient was started on levetiracetam for seizure prophylaxis, as well as intravenously (IV) administered acyclovir, ceftriaxone, vancomycin, and dexamethasone for suspected meningoencephalitis. Blood cultures were negative. CT chest demonstrated reticulonodular opacities and mediastinal lymphadenopathy (Figure 1). CT abdomen demonstrated hepatosplenomegaly. MRI brain with contrast demonstrated numerous, multifocal, ring-enhancing fluid collections throughout bilateral cerebral and cerebellar hemispheres with associated restricted diffusion on diffusion-weighted imaging (Figure 2). These were interpreted as multiple abscesses within the frontal, parietal, temporal, and occipital lobes and cerebellum bilaterally.

**Figure 3:**
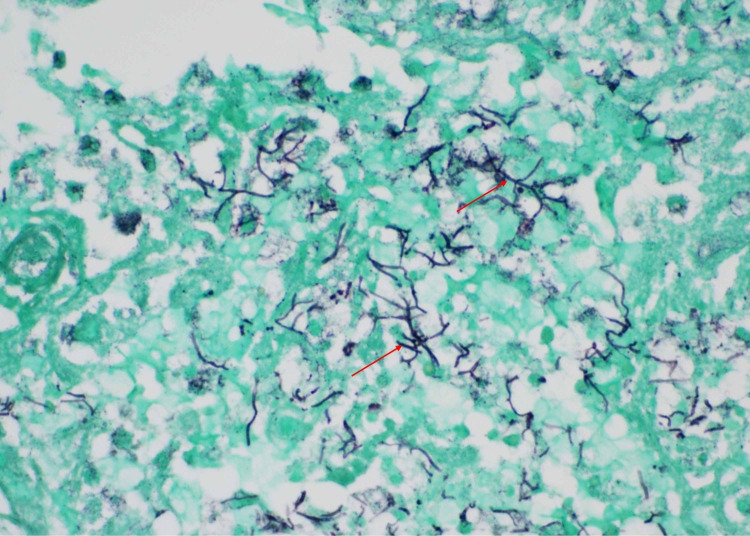
GMS stain taken at 600x magnification showing thin septate fungal hyphae, consistent with Nocardia spp Gomori methenamine silver (GMS) stain photomicrograph taken at 600x, demonstrating thin septate fungal hyphae, consistent with Nocardia spp.

Given the uncertainty of the diagnosis and newly identified symptomatic cerebral abscesses, the patient initially underwent a right frontal craniotomy with biopsy and aspiration of a lesion and a lumbar puncture. Cerebrospinal fluid (CSF) studies demonstrated 84% neutrophils, 14% lymphocytes, 2% monocytes, protein of 85 mg/dL, and glucose of 46 mg/dL. Initial aspirates grew *Nocardia otitidiscaviarum* (identified via matrix-assisted laser desorption ionization-time of flight mass spectrometry (MALDI-TOF MS)); the isolate was sent for antimicrobial susceptibility testing. Gomori methenamine silver (GMS) stain showed beaded branching filaments in brain tissue, consistent with invasive CNS nocardiosis (Figure 3). Antimicrobial agents were initially broadened to include coverage with imipenem and linezolid and adjusted to include IV amikacin (1200 mg/day), imipenem (three g/day), and trimethoprim/sulfamethoxazole (TMP/SMX; 15 mg/kg/day of the trimethoprim component). Cultures eventually speciated with *Nocardia otitidiscaviarum*, demonstrating susceptibilities to TMP/SMX, amikacin, tobramycin, and minocycline. As a result, linezolid was discontinued and minocycline (200 mg/day) was initiated.

**Figure 4:**
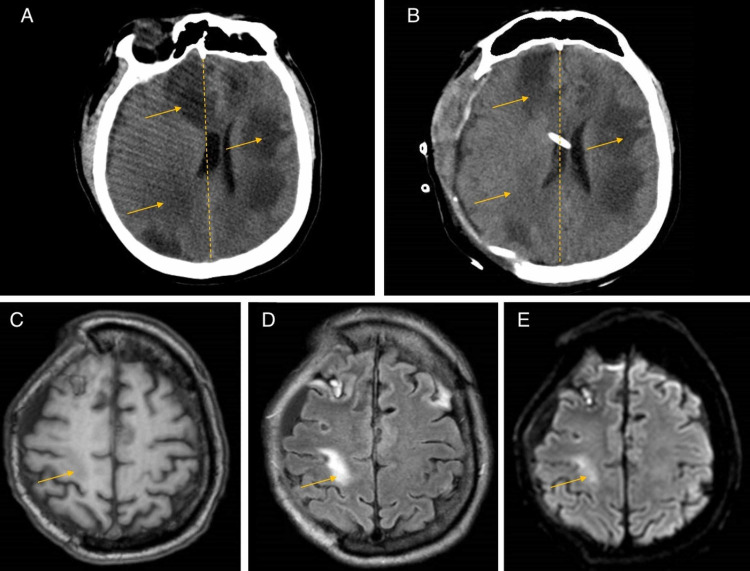
Peri-operative CT brain imaging and three-month postoperative MR brain imaging (A) Pre-operative CT head image demonstrating mass effect and midline shift more significant on the right side. (B) Postoperative CT head image after right decompressive hemicraniectomy. (C) Three-month postoperative T1-weighted post-contrast MR image demonstrating marked improvement of lesions. (D) Three-month postoperative T2-weighted-fluid-attenuated inversion recovery (T2-FLAIR) image further demonstrating an improvement of multifocal sites of confluent white matter, with the continuing resolution of vasogenic edema. (E) Three-month postoperative diffusion-weighted imaging (DWI) MR image demonstrating resolution of disease.

Approximately five days following the initiation of this treatment regimen, the patient’s mental status deteriorated. The patient became obtunded, not following commands, not moving all four extremities, and ultimately required intubation for airway protection. His neurologic examination further revealed anisocoria with a left nonreactive pupil, with cough and gag reflexes intact. CT scan of the head demonstrated enlargement of the multiple bilateral lesions with significant surrounding edema and mass effect worse on the right than the left (Figure 4). The patient was taken for a decompressive right hemicraniectomy with the placement of a right frontal external ventricular drain (EVD) for consideration of intrathecal therapy. In addition, aspiration of a larger abscess located in the right frontal lobe was performed.

In the setting of continued fluctuations in mental status as well as low EVD output, a trial of intrathecal amikacin was initiated. The patient received two total doses of 30 mg approximately 72 hours apart. Repeat CSF studies demonstrated no white blood cells, protein of less than 4 mg/dL, and glucose of 3 mg/dL. Intrathecal antibiotics were then discontinued, and the EVD was pulled. The patient’s mental status gradually improved. He completed a six-week course of combination therapy with IV amikacin, TMP/SMX, and minocycline and was then transitioned to dual therapy with oral TMP/SMX plus minocycline with a plan to continue for one year; following this, he will be transitioned to lifelong secondary prophylaxis with TMP/SMX. Follow-up MR imaging was done at one month and three months, demonstrating resolution of abscesses (Figure 4). A cranioplasty was completed approximately three months following the hemicraniectomy. Neurologic examination postoperatively was within normal limits overall aside from a moderate decrease in strength in his left upper extremity and proximal lower extremity. He was otherwise fully alert, oriented, without dysarthria or other focal deficits. He was ultimately discharged home for routine follow-up and physical therapy.

## Discussion

*Nocardia* is a genus representing a ubiquitous group of bacteria usually found in soil, organic matter, freshwater, and saltwater [1]. *Nocardia otitidiscaviarum* was first isolated from a guinea pig with ear disease in 1924 and has since been known as a rare opportunistic pathogen that causes primary cutaneous, lymphocutaneous, and pulmonary infections in immunocompromised patients as well as immunocompetent patients [11]. There were no published case reports of *Nocardia otitidiscaviarum *infection in humans until the mid-1960s, and it has been estimated that approximately 3% of *Nocardia* infections are due to this species.

The case in this report describes a rare presentation of multiple brain abscesses formed due to *Nocardia otitidiscaviarum* infection in an immunocompetent patient. Although several case reports of nocardial brain abscesses in immunocompetent hosts exist in the literature, these cases are due to *Nocardia* species that are more common such as *Nocardia abscessus* [5-6], *Nocardia farcinica* [7-8], and *Nocardia asteroides *[9-10]. Most of these cases involved a single abscess for which a craniotomy and aspiration were performed before a prolonged course of antibiotics. These immunocompetent patients improved significantly following treatment, with a resolution of neurologic symptoms and no recurrence of the disease.

Although a majority of extrapulmonary cases of nocardiosis occur in the CNS [2], there are only three reported cases of *Nocardia otitidiscaviarum* brain abscesses in immunocompetent hosts [12-14]. These relatively recent cases are outlined in Table 1, as is the case presented in this report. The cases in the literature involved varying antimicrobial regimens based on sensitivities, as well as varying surgical interventions, with only two cases to date reporting patient survival after prolonged hospital courses. One case involved a patient who died before surgical intervention [14]. Two other older case reports [15-16] also described patients with *Nocardia otitidiscaviarum *brain abscess who died as well. The primary neurosurgical intervention used in this case and most other cases [12-13], at least initially, was craniotomy with drainage of abscesses. This is standard also for the identification of the causative pathogen [17]. The case described in this report is the first case of *Nocardia otitidiscaviarum* brain abscess that necessitated hemicraniectomy. Although intrathecal antimicrobial agents have been delivered in other cases of brain abscess [17], this report also describes the first case where this technique was used for *Nocardia otitidiscaviarum* brain abscess specifically, with eventual resolution of disease. Although the course of intrathecal amikacin presented here was short, it is possible that direct intrathecal delivery of antibiotics expedited the treatment and resolution of intracranial disease.

**Table 1:** Treatment of Nocardia otitidiscaviarum brain abscess in immunocompetent patients TMP/SMX: trimethoprim/sulfamethoxazole; IV: intravenous; EVD: external ventricular drain

Case	Presentation	Past Medical History	Extent of Disease	Medical Management	Surgical Management	Outcome
Eren et al. (2016) [12]	A 69-year-old woman with 10 days of mild right hemiparesis.	None	Multiple bilateral hemispheric lesions on MRI.	Initially meropenem (6 g/day) and amikacin (1 g/day), then switched to meropenem (6 g/day) and TMP/SMX (3600 mg/day). After eight weeks of IV antimicrobial therapy, the patient was discharged on oral TMP/SMX.	Lesions were drained by stereotactic craniotomy once a week consecutively for three weeks.	Regression of lesions confirmed at 1-year MRI.
Ishihara et al. (2014) [13]	A 79-year-old woman with five days of mild left hemiparesis and progressive amnesia.	History of tuberculosis in her 20s.	A single ring-enhancing lesion in the right frontal lobe with surrounding edema on MRI.	Initially managed with meropenem (6 g/day), sulbactam/ampicillin (3 g/day), and cefozopran (2 g/day) before surgical intervention. After the second abscess drainage, the patient was switched to oral TMP/SMX.	The lesion was aspirated twice via craniotomy.	Resolution of lesion though with persisting anosognosia and unilateral spatial neglect.
Pelaez et al. (2009) [14]	An 85-year-old woman who was initially admitted for dyspnea, cough, and pleuritic chest pain.	History of chronic obstructive pulmonary disease (COPD), coronary artery disease, and hypertension.	CT of the chest demonstrated multiple pulmonary nodules, and CT of the brain demonstrated a nodular lesion in the left frontotemporal lobe.	Initial management involved IV TMP/SMX (2400/480 mg/day), and imipenem (4 g/day), which was then switched to linezolid (600 mg twice/day) and dexamethasone (40 mg/day).	The patient died before surgical intervention.	The patient died.
Einstein et al. (2020)	A 46-year-old man admitted for new-onset generalized tonic-clonic seizure and altered mental status.	History of well-controlled type 2 diabetes.	CT chest demonstrated multiple reticulonodular opacities and mediastinal lymphadenopathy. MRI brain demonstrated numerous ring-enhancing lesions in bilateral cerebral and cerebellar hemispheres.	Initial management involved Linezolid (1200 mg/day), TMP/SMX (900 mg/day), imipenem, and amikacin (1200 mg/day). After susceptibilities were obtained, minocycline (200 mg/day) was started, and imipenem was discontinued. The patient also received two total doses of 30 mg amikacin intrathecally. Six weeks of IV antibiotics treatment was followed by 1 year of oral treatment with minocycline and TMP/SMX.	Initial management involved craniotomy and biopsy, followed by right hemicraniectomy and EVD placement.	Regression of lesions and resolution of altered mental status.

As for antimicrobial therapy, each reported case used a variety of broad-spectrum agents prior to obtaining susceptibilities (Table 1). *Nocardia otitidiscaviarum* is typically susceptible to kanamycin, gentamicin, amikacin, sulfamethoxazole, and ciprofloxacin [11]. It is generally resistant to ceftriaxone, ampicillin, amoxicillin-clavulanate, carbenicillin, and imipenem; it is often resistant to all beta-lactam antibiotics [11]. In the case described in this report, the species was susceptible to TMP/SMX, linezolid, amikacin, tobramycin, and minocycline, with resistance to imipenem, amoxicillin-clavulanate, ceftriaxone, ciprofloxacin, moxifloxacin, and clarithromycin. This led to a final regimen of amikacin, TMP/SMX, and minocycline. 

Nocardiosis can be challenging to definitively diagnose, but it should be considered in a differential diagnosis in the setting of multiorgan disease especially involving the lungs and CNS. Broad-spectrum coverage should include an aminoglycoside such as amikacin, as well as TMP/SMX. Intrathecal amikacin could also be considered, as it was effective in our case as described.

## Conclusions

In conclusion, *Nocardia otitidiscaviarum* brain abscess is a rare disease that has led to several fatalities. In standard fashion, a combination of IV antimicrobial agents and craniotomy with aspiration can be used as treatment. However, to prevent further neurologic decompensation and demise, it may be necessary to perform hemicraniectomy, as in our case. There may also be a role for intrathecal antibiotic therapy, potentially with amikacin as a primary agent. More cases will be needed to develop a more protocolized treatment for this specific disease. However, rapid identification of the pathogen, use of broad-spectrum antibiotics initially, understanding of antimicrobial susceptibilities, and timely neurosurgical intervention are clearly critical in ensuring the best possible patient outcome.
